# Detecting Novel Genetic Variants Associated with Isoniazid-Resistant *Mycobacterium tuberculosis*

**DOI:** 10.1371/journal.pone.0102383

**Published:** 2014-07-15

**Authors:** Sandhya Shekar, Zhen Xuan Yeo, Joshua C. L. Wong, Maurice K. L. Chan, Danny C. T. Ong, Pumipat Tongyoo, Sin-Yew Wong, Ann S. G. Lee

**Affiliations:** 1 Division of Medical Sciences, National Cancer Centre, Singapore, Singapore; 2 Department of Infectious Diseases, Singapore General Hospital, Singapore, Singapore; 3 Office of Clinical & Academic Faculty Affairs, Duke-NUS Graduate Medical School, Singapore, Singapore; 4 Department of Physiology, Yong Loo Lin School of Medicine, National University of Singapore, Singapore, Singapore; Institut de Génétique et Microbiologie, France

## Abstract

**Background:**

Isoniazid (INH) is a highly effective antibiotic central for the treatment of *Mycobacterium tuberculosis* (MTB). INH-resistant MTB clinical isolates are frequently mutated in the *katG* gene and the *inhA* promoter region, but 10 to 37% of INH-resistant clinical isolates have no detectable alterations in currently known gene targets associated with INH-resistance. We aimed to identify novel genes associated with INH-resistance in these latter isolates.

**Methodology/Principal Findings:**

INH-resistant clinical isolates of MTB were pre-screened for mutations in the *katG*, *inhA*, *kasA* and *ndh* genes and the regulatory regions of *inhA* and *ahpC*. Twelve INH-resistant isolates with no mutations, and 17 INH-susceptible MTB isolates were subjected to whole genome sequencing. Phylogenetically related variants and synonymous mutations were excluded and further analysis revealed mutations in 60 genes and 4 intergenic regions associated with INH-resistance. Sanger sequencing verification of 45 genes confirmed that mutations in 40 genes were observed only in INH-resistant isolates and not in INH-susceptible isolates. The ratios of non-synonymous to synonymous mutations (dN/dS ratio) for the INH-resistance associated mutations identified in this study were 1.234 for INH-resistant and 0.654 for INH-susceptible isolates, strongly suggesting that these mutations are indeed associated with INH-resistance.

**Conclusion:**

The discovery of novel targets associated with INH-resistance described in this study may potentially be important for the development of improved molecular detection strategies.

## Introduction

*Mycobacterium tuberculosis* (MTB) is a leading cause of mortality globally, with about 8.7 million new cases of tuberculosis and 1.4 million deaths reported in 2011 [1], with the emergence of multidrug-resistant (MDR) tuberculosis further hampering the control of the disease. Drug resistance in MTB develops when random naturally occurring chromosomal mutations occur in genes encoding a drug target or a drug-activating enzyme, and the subsequent selection of these mutants when there is incomplete suppression of growth, typically when patients have poor adherence to the therapeutic regimen. It has been estimated that in 2011, there were 630,000 cases of MDR TB [1], defined as strains of TB that are resistant to isoniazid (INH) and rifampin.

INH is a highly effective anti-tuberculosis drug, central for the treatment of MTB. The mode of action of INH is to inhibit mycolic acid synthesis [2]. The complex cell envelope of MTB protects the bacterium from antibiotics, oxidative stress and toxic macromolecules, and is composed of a plasma membrane at the base, and a thick outer layer that includes complex lipids such as mycolic acid and phthiocerol dimycocerosate (PDIM) [3]–[5].

INH is a prodrug activated by the catalase-peroxidase enzyme, KatG [6]. The majority of resistance-associated mutations occur in the *katG* gene, with mutations resulting in diminished activation of INH [6]. Other important resistance-conferring mutations are promoter or structural mutations in *inhA*, *ahpC*, *kasA* and *ndh* [7]–[11]. However, many of the mutations detected in these genes have been also observed in INH-susceptible isolates and/or in association with *katG* mutations [2], [12]–[16].

Between 10 to 37% of INH-resistant clinical isolates have no detectable alterations in any of the currently known gene targets [7], [15], [16], suggesting that there are other loci involved in INH resistance that have yet to be identified. To date, none of the studies that performed genome sequencing on drug-resistant MTB have focussed on INH resistance [17]–[20]. Our aim was to identify novel genes associated with INH resistance by sequencing the whole genomes of INH-resistant clinical isolates of MTB with no detectable mutations in the known genes associated with INH resistance, and to validate the identified candidate genes in an independent set of MTB strains. Several novel genes associated with INH resistance are described.

## Materials and Methods

### MTB Isolates and DNA Extraction

Clinical isolates of MTB were from the Central Tuberculosis Laboratory, Department of Pathology, Singapore General Hospital. Phenotypic drug susceptibility testing was done with the BACTEC 460 system (Becton Dickinson, Towson, MD), as previously described [14]. The BACTEC system is a well recognised method for susceptibility testing, and uses radiometric technology for the rapid, qualitative detection of mycobacterial growth in the presence of isoniazid, tested at a concentration of 0.1 ug/ml. DNA was extracted from bacterial colonies cultured on Lowenstein-Jensen slants by heat-inactivation, digestion with lysozyme and proteinase K, followed by precipitation of the nucleic acids, as described previously [21], [22].

### Whole-Genome Sequencing

All INH-resistant isolates had previously been screened for known mutations found in *katG*, *inhA*, *kasA*, *ndh* genes, and the regulatory regions of *inhA* and *ahpC*, and had no detectable mutations [7], [14], [23]. Whole-genome sequencing was done in two stages. In the first stage, DNA from 12 INH-resistant and 6 INH-susceptible isolates was sequenced on the Applied Biosystems SOLiD 3 plus system by Mission Biotech Co., Ltd. Taiwan and AITBIOTECH Pte Ltd., Singapore. However, preliminary Sanger sequencing analysis showed that many of the mutations detected in INH-resistant isolates were also found in INH-susceptible isolates (data not shown). In the second stage, DNA from 11 additional INH-susceptible isolates were subjected to whole-genome sequencing to facilitate the selection of INH-resistant specific mutations, and these were sequenced using the Illumina HiSeq2000 Sequencing System by 1^st^ Base Pte Ltd, Singapore.

### Mapping Assembly and Mutation Detection

SOLiD 3 single-end reads and Illumina paired-end reads were aligned against *Mycobacterium tuberculosis* H37Rv (GenBank accession no. AL123456.2) using BWA 0.5.9, which takes into account read quality during alignment, resulting in low quality reads not being aligned [24]. Mutations were detected using GATK (version 1.3-24-gc8b1c92), whereby the BAM alignment file was pre-processed by local realignment and de-duplication [25]. Subsequently, variant calling was performed using UnifiedGenotyper and VariantFiltration from GATK against the pre-processed BAM file. Mutations detected in all isolates with <50% allele frequency were filtered. Only mutations that were specific to INH-resistant isolates were selected for subsequent analyses. Some INH-resistant specific mutations could be incorrectly detected if coverage in susceptible isolates is low (less than 4×), thus only mutations with ≥4× coverage (at the mutation site) in at least one susceptible isolate were retained. ANNOVAR was used to annotate mutations by gene name, mutation type and genomic features [26].

### Phylogenetic Analysis

**Figure 1:**
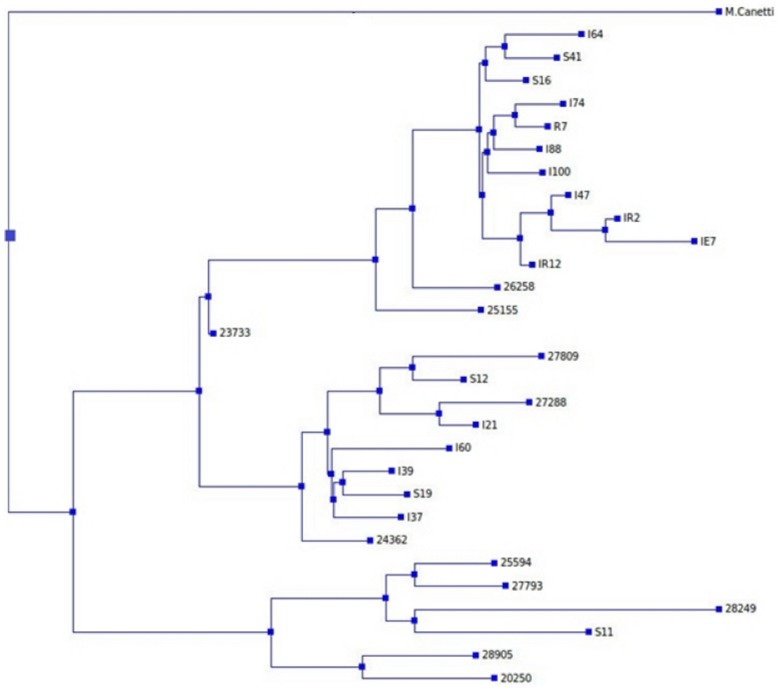
Phylogenetic tree of 29 Mycobacterium tuberculosis isolates. The whole genome sequences of the 29 isolates were used to construct the tree, with *Mycobacterium canetti* used as an outlier, in order to identify and eliminate mutations that were phylogenetically related. Drug-resistant isolates were coded according to the drugs they were resistant to: isolates resistant to isoniazid were coded with “I”; those resistant to rifampicin, “R”; those resistant to streptomycin, “S”; those resistant to ethambutol, “E”. All isolates coded with 5-digit numbers (2XXXX), were INH-susceptible isolates. Spoligotyping assigned the isolates into these lineages: I64, S41, S16, I74, R7, I88, I100, I47, IR2, IE7 and IR12 belonged to the Beijing lineage; 26258, 25155, 23733, I37 and 27793, the U lineage; 27809, 27288, I21, I39, S19, 24362, 25594 and 28249, the T lineage; S12, the Haarlem lineage; I60, the LAM lineage; 28905, the H37Rv lineage; and the isolates S11 and 20250, the EAI lineage.

Phylogenetically related mutations could affect the accuracy in selecting the INH resistance associated genes and intergenic regions. Phylogenetic analysis of 29 isolates that were whole genome sequenced was carried out using TreeBeST, which uses a maximum likelihood approach for phylogenetic tree construction. Figure 1 shows the phylogenetic tree constructed with *Mycobacterium canetti* as an outlier [27].

Filtering of phylogenetically related mutations was performed in the following way, as has been described previously in [17]:

- mutations that were present only within one single clade of the tree (Figure 1) were removed;
- mutations that were present in two or more subclades (Figure 1) and in which there was an INH-susceptible isolate, were removed. The remaining set of 4229 variants was used to identify INH- resistance associated genes and intergenic regions.

### Identification of INH resistance associated mutations within coding regions and intergenic regions

**Figure 2:**
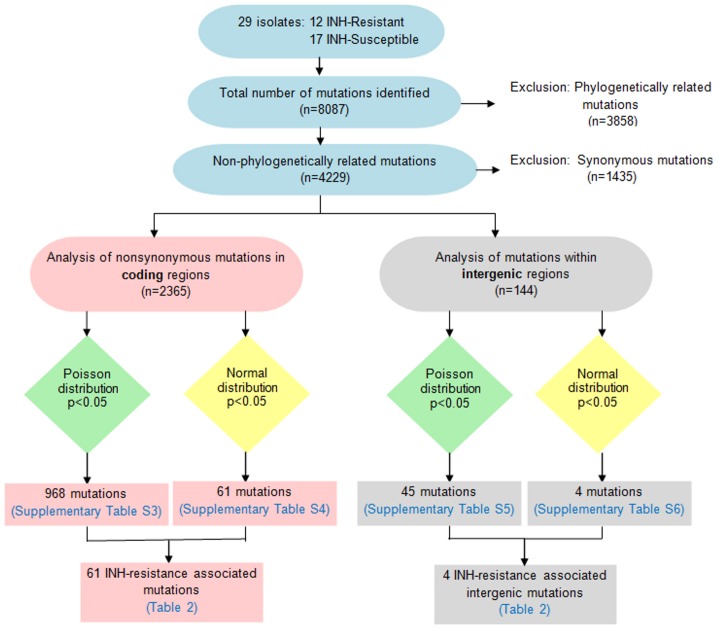
Flowchart showing the steps used for identifying INH-resistance associated genes and intergenic regions. The whole genome sequences of 29 Mycobacterium tuberculosis isolates were aligned with the reference genome H37Rv (GenBank accession no. AL123456.2) and mutations in the sequences were identified. Phylogenetic analysis was carried out to eliminate phylogenetically related mutations. The Poisson and Normal distributions were used to identify mutations associated with INH-resistance.

Mutations associated with INH-resistance were identified using the approach described previously by Zhang et al. [17]. In brief, the Poisson distribution (*p*<0.05) was used to identify mutations in coding and intergenic regions, not occurring by chance. The Normal distribution (*p*<0.05) was used to select mutations that were present in a higher proportion in INH-resistant isolates than in INH-susceptible isolates (Figure 2).

### dN/dS Calculation

To confirm our findings, the ratio of non-synonymous to synonymous mutations (dN/dS ratio) of the INH-resistance associated genes as well as whole genome sequences of both INH-resistance and INH-susceptible isolates was determined [28]. The ancestral sequences for the dN/dS ratio calculation was found using PAML [29]. The dN/dS ratio was calculated using the KaKs Calculator [30].

### Sanger Sequencing

Specific primers were designed to amplify regions of ∼300 to 600 base pairs flanking mutations detected from whole-genome sequencing (Table S1). Polymerase chain reaction (PCR) amplification was performed using HotStar *Taq* polymerase (Qiagen, USA) and the PCR products were purified with FastAP (Fermentas, Canada) and Exonuclease I enzyme (Fermentas), according to the manufacturer's instructions. Sanger sequencing was performed using the standard dye terminator chemistry (BigDye Terminator v3.1, Applied Biosystems, USA), and analysed on a 3130*xl* Genetic Analyser (Applied Biosystems). Sequencing results were aligned to the MTB H37Rv sequence (GenBank accession no. AL123456) using SeqMan Pro Lasergene 8 software (DNASTAR, Wisconsin, USA).

### Data Access

Raw sequence data has been submitted to the European Nucleotide Archive (http://www.ebi.ac.uk/ena/) under accession number ERP001993.

## Results

### Mutations detected by Whole-Genome Sequencing

To identify INH-resistance specific mutations, we performed whole-genome sequencing for twelve INH-resistant MTB isolates (n = 12) with no detectable mutations in the *inhA*, *kasA* and *ndh* genes, codon 315 of *katG* and the promoter regions of *inhA* and *ahpC*. Nine of these twelve INH-resistant isolates were mono-resistant to INH. Whole-genome sequencing of 17 INH-susceptible isolates was also done to allow for the discrimination of resistance-associated mutations (Table S2).

The average depth of coverage for the twelve INH-resistant MTB isolates was 33-fold (19- to 41-fold), with an average of 86% of bases with at least 8-fold coverage. For the 17 INH-susceptible MTB isolates, the average depth of coverage was 534-fold, and an average of 94% bases had at least 8-fold coverage. As whole-genome sequencing had been performed using two sequencing platforms, the 11 INH-susceptible isolates sequenced in the second stage had higher read coverage, as the more advanced Illumina HiSeq2000 Sequencing System was utilized. The overall higher coverage for INH-susceptible MTB isolates resulted in a higher specificity in detecting INH-resistant specific mutations.

In total, we detected 8087 mutations of which 1054 were found only in INH-resistant strains (Table 1). Of these 1054 mutations, 289 were synonymous, 565 were non-synonymous, 9 were nonsense, and 65 were insertions or deletions. Thus, a total of 639 non-synonymous and nonsense mutations, insertions and deletions were identified in INH-resistant strains (Table S2).

**Table 1:** Variants identified by whole genome sequencing of 12 isoniazid-resistant and 17 isoniazid-susceptible *Mycobacterium tuberculosis* isolates.

Mutation type	Resistant and Susceptible isolates*	Resistant isolates only	Susceptible isolates only
**All (n = 8087)**	**1949**	**1054**	**5084**
**Coding**	**1669**	**928**	**4392**
Synonymous	573	289	1552
Nonsynonymous	977	565	2397
Nonsense	14	9	57
Insertions/Deletions	105	65	386
**Non-coding (intergenic)**	**280**	**126**	**692**

* Variants that can be found in both isoniazid-resistant and -susceptible isolates.

### Genes and intergenic regions associated with INH resistance

A total of 2,365 non-synonymous and 144 intergenic variants were identified in INH-resistant and INH-susceptible strains, after excluding phylogenetically related and synonymous mutations (Figure 2). We further excluded potential random and non-resistance specific variations (Figure 2). This resulted in 61 non-synonymous mutations in 60 genes (Tables 2, S3 and S4) and 4 intergenic variations (Tables 2, Tables S5 and S6) selected as candidates associated with INH-resistance.

**Table 2:** Genes and intergenic regions potentially associated with INH-resistance in *M. tuberculosis*.

Gene Name	Rv No.	Functional Category*	Function*
*-*	*Rv0175*	Cell wall and cell processes	Unknown
*-*	*Rv0236c*	Cell wall and cell processes	Biosynthesis of the mycobacterial Cell wall
*pstS2*	*Rv0932c*	Cell wall and cell processes	Active transport of inorganic phosphate across the membrane; required for binding-protein-mediated phosphate transport
*mscL*	*Rv0985c*	Cell wall and cell processes	Regulation of osmotic pressure changes within the cell
*-*	*Rv0987*	Cell wall and cell processes	Active transport of adhesion component across the membrane; translocation of the substrate across the membrane
*esxL*	*Rv1198*	Cell wall and cell processes	Unknown
*-*	*Rv1362c*	Cell wall and cell processes	Unknown
*-*	*Rv1877*	Cell wall and cell processes	Unknown; possibly involved in transport of drug across the membrane.
*-*	*Rv2576c*	Cell wall and cell processes	Unknown
*-*	*Rv2869c*	Cell wall and cell processes	Controls membrane composition
*dacB2*	*Rv2911*	Cell wall and cell processes	Peptidoglycan synthesis
*lppY*	*Rv2999*	Cell wall and cell processes	Unknown
*lytB1*	*Rv3382c*	Cell wall and cell processes	Unknown; possibly involved in drug/antibiotic tolerance
*-*	*Rv3448*	Cell wall and cell processes	Unknown; possibly involved in transport across the membrane
*-*	*Rv0194*	Cell wall and cell processes	Active transport of drugs across the membrane; energy coupling to the transport system and for the translocation of the substrate across the membrane
*hycQ*	*Rv0086*	Metabolism and respiration	Involved in hydrogen metabolism
*-*	*Rv0338c*	Metabolism and respiration	Unknown; probably involved in cellular metabolism
*-*	*Rv0517*	Metabolism and respiration	Unknown; probably involved in cellular metabolism
*-*	*Rv0793*	Metabolism and respiration	Unknown
*fprB*	*Rv0886*	Metabolism and respiration	Electron transfer protein
*eno*	*Rv1023*	Metabolism and respiration	Glycolysis
*moeY*	*Rv1355c*	Metabolism and respiration	Biosynthesis of a demolybdo cofactor (molybdopterin), necessary for molybdoenzymes; activation of the small subunit of the molybdopterin converting factor (MOAD)
*frdD*	*Rv1555*	Metabolism and respiration	Interconversion of fumarate and succinate
*gnd1*	*Rv1844c*	Metabolism and respiration	Involved in hexose monophosphate shunt
*ureC*	*Rv1850*	Metabolism and respiration	Conversion of urea to NH3
*-*	*Rv2296*	Metabolism and respiration	Converts haloalkanes to corresponding alcohol and halides
*pca*	*Rv2967c*	Metabolism and respiration	Gluconeogenesis and lipogenesis
*atsB*	*Rv3299c*	Metabolism and respiration	Sulfate and phenol generation from phenol sulfate
*-*	*Rv3401*	Metabolism and respiration	Unknown; probably enzyme involved in cellular metabolism.
*-*	*Rv3537*	Metabolism and respiration	Probably involved in cellular metabolism; predicted to be involved in lipid catabolism
*-*	*Rv0104*	Hypothetical protein	Unknown
*-*	*Rv0574c*	Hypothetical protein	Unknown
*-*	*Rv1069c*	Hypothetical protein	Unknown
*-*	*Rv1118c*	Hypothetical protein	Unknown
*-*	*Rv1504c*	Hypothetical protein	Unknown
*-*	*Rv1896c*	Hypothetical protein	Unknown
*-*	*Rv1977*	Hypothetical protein	Unknown
*-*	*Rv2184c*	Hypothetical protein	Unknown
*-*	*Rv2432c*	Hypothetical protein	Unknown
*-*	*Rv2917*	Hypothetical protein	Unknown
*-*	*Rv2955c*	Hypothetical protein	Unknown
*-*	*Rv3181c*	Hypothetical protein	Unknown
*fadE1*	*Rv0131c*	Lipid metabolism	Unknown; but involved in lipid degradation
*gpsA*	*Rv0564c*	Lipid metabolism	Phospholipid biosynthesis
*-*	*Rv0726c*	Lipid metabolism	Possible methyltransferase
*pks5*	*Rv1527c*	Lipid metabolism	Polyketide metabolism
*-*	*Rv1729c*	Lipid metabolism	Possible methyltransferase
*mbtB*	*Rv2383c*	Lipid metabolism	Biogenesis of the hydroxyphenyloxazoline-containing siderophore mycobactins
*mbtA*	*Rv2384*	Lipid metabolism	Biogenesis of the hydroxyphenyloxazoline-containing siderophore mycobactins
*cmaA1*	*Rv3392c*	Lipid metabolism	Cyclopropane function
*-*	*Rv3480c*	Lipid metabolism	May be involved in synthesis of triacylglycerol
*rpoB*	*Rv0667*	Information pathways	Catalyzes the transcription of DNA into RNA
*sigI*	*Rv1189*	Information pathways	Promotes attachment of the RNA polymerase to specific initiation sites
*-*	*Rv3649*	Information pathways	Helicase activity
*PPE8*	*Rv0355c*	PE/PPE#	Unknown
*PE_PGRS7*	*Rv0578c*	PE/PPE#	Unknown
*PPE24*	*Rv1753c*	PE/PPE#	Unknown
*-*	*Rv0094c*	Insertion seqs and phages	Unknown
*-*	*Rv2659c*	Insertion seqs and phages	Integration of a phage into the host genome by site-specific recombination
*-*	*Rv1358*	Regulatory proteins	Involved in transcriptional mechanism
*-*	*Rv0835-Rv0836c* ∧	-	-
*-*	*Rv1068c-Rv1069c* ∧	-	-
*-*	*Rv3812-Rv3813c* ∧	-	-
*-*	*Rv3822-Rv3823c* ∧	-	-

* Functional Category and Functions of genes are retrieved from Tuberculist [39] (http://tuberculist.epfl.ch/).

# Proteins whose N-termini contain the characteristic motifs Pro-Glu (**PE**) or Pro-Pro-Glu (**PPE**).

∧ Intergenic regions.

### dN/dS ratios

Table 3 shows the dN/dS ratio for INH-resistant and INH-susceptible isolates for the whole genome and for the 60 genes associated with INH resistance identified in this study. The dN/dS ratios for INH-resistant and INH-susceptible isolates were 1.234 and 0.654 respectively for the 60 genes associated with INH resistance.

**Table 3:** The ratio of non-synonymous to synonymous mutations (dN/dS) in INH-resistant (n = 12) and INH-susceptible (n = 17) isolates, in (1) whole genome sequences and in (2) 60 INH-resistance associated genes.

	INH-resistant isolates	INH-susceptible isolates
Whole Genome	0.794	0.766
60 genes	1.234	0.654

### INH-resistant mutations verified by Sanger sequencing

To determine if the mutations identified from whole genome sequencing occurred only in INH-resistant isolates and not in INH-susceptible isolates, verification was done on additional INH-resistant and INH-susceptible isolates by Sanger sequencing. For each mutation, the number of isolates used for verification by Sanger sequencing is shown in Supplementary Table S7. Importantly, the majority of these mutations were detected in additional INH-resistant isolates (column J, Table S7), suggesting that these could be high-confidence mutations. Of the 45 genes screened for mutations, only 5 (*gpsA*, *gnd1*, *atsB*, Rv1069c and Rv2869c) had mutations in INH-susceptible isolates (Table S7). A list of all the INH-resistant and INH-susceptible isolates used for verification is provided in Table S8.

## Discussion

This is the first study to our knowledge that has whole-genome sequenced INH-resistant isolates with no mutations in known regions associated with INH resistance. Although there are several recent reports in the literature on MTB genomes, these studies did not sequence MTB isolates pre-screened for mutations in INH-resistance associated genes [17]–[20], [31]–[35].

Recently, two landmark studies employed genome sequencing of *M. tuberculosis* to identify genes and intergenic regions associated with drug resistance, but did not focus on INH resistance. Zhang *et al.* [17] performed genome sequencing on multi-drug resistant (MDR) and extensively drug-resistant (XDR) isolates, while Farhat and colleagues [20] sequenced a combination of epidemiologically linked, non-epidemiologically linked, drug-resistant and drug-sensitive MTB isolates. Our work described here is thus novel. In particular the unique approach used, i.e. the pre-screening of *M. tuberculosis* isolates to select isolates with no detectable alterations in known INH resistance genes, has resulted in the identification of several novel genes and intergenic regions associated with INH-resistance.

Of the 60 genes identified as INH-resistance associated, 12 were hypothetical proteins of unknown functions, 15 had annotated functions in cell wall and cell processes, 15 were associated with metabolism and respiration, and 9 were genes involved in lipid metabolism. This current study and two recent ones have identified members of the polyketide synthase family in drug-resistant MTB isolates [17], [20]. Polyketide synthases are involved in complex lipid biosynthesis and assembly [5]. The *pks5* gene is a mas-like polyketide synthase gene; MTB with *pks5* mutants introduced into the lungs of mice had lower rates of multiplication as compared to wild-type strains [36]. Hence mutations in *pks* genes may be associated with INH-resistance and further functional validation is warranted.

Mutations within intergenic regions such as the regulatory (promoter) regions of *inhA* and *ahpC* are associated with INH-resistance. Recent genome sequencing of *M. tuberculosis* have identified additional drug resistance-associated intergenic regions [17], [20]. For example, the newly discovered intergenic regions, *thyA-Rv2765* and *thyX-hsdS.1*, were shown to increase the levels of gene expression of flanking genes as compared to non-mutant controls *in vitro*, providing functional evidence that these regions may be important in drug resistance [17].

The ratio of non-synonymous mutations to synonymous mutations (dN/dS) suggests the rate of evolution of genes and such rates play a major role in identifying potential drug targets [37]. In the case of positive selection, genes with dN/dS ratio >1 have adapted themselves such that they become fit to reproduce themselves against drugs that act on them. This current work has identified genes that have dN/dS ratio of 1.234 in INH-resistant isolates, in contrast to a dN/dS ratio of 0.654 in INH-susceptible isolates, strongly suggesting that mutations in these genes are indeed associated with INH-resistance.

One limitation of this study is that the small number of isolates that were whole-genome sequenced (12 INH-resistant and 17 INH-susceptible isolates) may have led to the identification of only some INH-resistant mutations, which occur in very low frequencies. It is anticipated that whole genome sequencing of more MTB isolates could potentially reveal additional novel mutations associated with INH-resistance. In addition, due to the limited number of clinical isolates in this study, statistical confirmation that the INH-resistance associated mutations are indeed linked to the INH-resistant phenotype, has not been shown.

Another limitation of this study is that cloning of each mutant allele and determination of their functional effect in INH resistance has not been performed. Functional analysis will be required to conclusively show that each of these mutations is associated with INH resistance. Allelic exchange has also been proposed as an approach for validating the effect of mutations associated with antibiotic resistance [38]. However this approach will involve the culture of MTB in BSL-3 laboratories.

In conclusion, the search for identification of the additional genes associated with INH-resistance as described in this current study, will be important for the development of comprehensive molecular detection strategies, more efficient than current susceptibility testing methods based on culture. Such molecular screening methods could aid in more appropriate treatment given earlier to patients and have the potential to decrease transmission of the resistant strains. In addition, the discovery of the new loci reported here may reveal novel targets suitable for the development of alternative therapeutic options.

## Supporting Information

Table S1 **List of primers used for PCR amplification and Sanger sequencing.**
(XLSX)

Table S2 **List of nonsynonymous and nonsense mutations, insertions and deletions identified by whole genome sequencing in INH-resistant isolates.**
(XLSX)

Table S3 **List of nonsynonymous, stoploss and stopgain mutations with Poisson distribution p-value<0.05.**
(XLSX)

Table S4 **List of nonsynonymous, stoploss and stopgain mutations with Normal distribution p-value<0.05.**
(XLSX)

Table S5 **List of intergenic mutations with Poisson distribution p-value<0.05.**
(XLSX)

Table S6 **List of intergenic mutations with Normal distribution p-value<0.05.**
(XLSX)

Table S7 **List of mutations verified using Sanger sequencing.**
(XLSX)

Table S8 **Drug susceptibility of 101 INH-resistant isolates & 99 INH-susceptible isolates tested.**
(XLSX)
